# IFNAR1 Deficiency Impairs Immunostimulatory Properties of Neutrophils in Tumor-Draining Lymph Nodes

**DOI:** 10.3389/fimmu.2022.878959

**Published:** 2022-06-27

**Authors:** Timon Hussain, Maksim Domnich, Sharareh Bordbari, Ekaterina Pylaeva, Elena Siakaeva, Ilona Spyra, Irem Ozel, Freya Droege, Anthony Squire, Stefan Lienenklaus, Kathrin Sutter, Anja Hasenberg, Matthias Gunzer, Stephan Lang, Jadwiga Jablonska

**Affiliations:** ^1^ Department of Otorhinolaryngology, University Hospital Essen, University Duisburg-Essen, Essen, Germany; ^2^ Institute for Experimental Immunology and Imaging, University Duisburg-Essen, Essen, Germany; ^3^ Institute for Laboratory Animal Science, Institute of Immunology, Hannover Medical School, Hannover, Germany; ^4^ Institute for Virology, University Hospital Essen, University Duisburg-Essen, Essen, Germany; ^5^ Biospectroscopy Research Department, Institut für Analytische Wissenschaften (ISAS) e.V., Dortmund, Germany; ^6^ German Cancer Consortium (DKTK) partner site Düsseldorf/Essen, Essen, Germany

**Keywords:** IFNAR1, Interferons, neutrophils, tumor-draining lymph nodes, neutrophils/T-cells interactions, intravital microscopy, HNC (head and neck cancer)

## Abstract

Tumor-draining lymph nodes (TDLNs) are the first organs where the metastatic spread of different types of cancer, including head and neck cancer (HNC), occurs and have therefore high prognostic relevance. Moreover, first anti-cancer immune responses have been shown to be initiated in such LNs *via* tumor-educated myeloid cells. Among myeloid cells present in TDLNs, neutrophils represent a valuable population and considerably participate in the activation of effector lymphocytes there. Tumor-supportive or tumor-inhibiting activity of neutrophils strongly depends on the surrounding microenvironment. Thus, type I interferon (IFN) availability has been shown to prime anti-tumor activity of these cells. In accordance, mice deficient in type I IFNs show elevated tumor growth and metastatic spread, accompanied by the pro-tumoral neutrophil bias. To reveal the mechanism responsible for this phenomenon, we have studied here the influence of defective type I IFN signaling on the immunoregulatory activity of neutrophils in TDLNs. Live imaging of such LNs was performed using two-photon microscopy in a transplantable murine HNC model. Catchup^IVM-red^ and *Ifnar1^-/-^* (type I IFN receptor- deficient) Catchup^IVM-red^ mice were used to visualize neutrophils and to assess their interaction with T-cells *in vivo*. We have evaluated spatiotemporal patterns of neutrophil/T-cell interactions in LNs in the context of type I interferon receptor (IFNAR1) availability in tumor-free and tumor-bearing animals. Moreover, phenotypic and functional analyses were performed to further characterize the mechanisms regulating neutrophil immunoregulatory capacity. We demonstrated that inactive IFNAR1 leads to elevated accumulation of neutrophils in TDLNs. However, these neutrophils show significantly impaired capacity to interact with and to stimulate T-cells. As a result, a significant reduction of contacts between neutrophils and T lymphocytes is observed, with further impairment of T-cell proliferation and activation. This possibly contributes to the enhanced tumor growth in *Ifnar1^-/-^* mice. In agreement with this, IFNAR1-independent activation of downstream IFN signaling using IFN-λ improved the immunostimulatory capacity of neutrophils in TDLNs and contributed to the suppression of tumor growth. Our results suggest that functional type I IFN signaling is essential for neutrophil immunostimulatory capacity and that stimulation of this signaling may provide a therapeutic opportunity in head and neck cancer patients.

## Introduction

In the context of cancer, neutrophils can exhibit both pro- and anti-tumoral properties. Their behavior is determined by signals from tumor cells and the tumor microenvironment (1–3). In many cancer entities, including head and neck cancer (HNC), high intra-tumoral neutrophil counts have been shown to be associated with a worse patient prognosis (4–6). Several responsible mechanisms have been identified, including the support of angiogenesis (7, 8), the promotion of metastatic spread (9–12), the regulation of B cell activation (13, 14), as well as the regulation of T-cell responses in tumor tissue (15–17). Neutrophil tumorigenicity is regulated by tumor-derived factors, such as type I interferons (IFNs) (2, 8, 18, 19), transforming growth factor beta (TGFβ) (20), or granulocyte-colony-stimulating factor (G-CSF) (21). While TGFβ and G-CSF have been shown to stimulate pro-tumoral activity of neutrophils, type I IFNs promote anti-tumoral functions of such cells. Of note, type I IFN signaling does not only affect the anti-tumoral differentiation of these cells, but has also been shown to stimulate anticancer immunosurveillance (22, 23) as well as negatively affecting the metastatic dissemination of solid tumors *via* a variety of mechanisms.

Among pathways by which neutrophils affect tumor progression, the regulation of adaptive immune responses has recently been investigated in more detail. In the immediate tumor environment, neutrophils were shown to directly stimulate anti-tumor responses by presenting antigens to CD4^+^ T-cells and as well as activating cytotoxic CD8^+^ T-cells (16, 18, 24). On the other side of the spectrum, pro-tumoral neutrophils can suppress cytotoxic CD8^+^ T lymphocytes (25) or secrete chemo-attractants such as CCL17 to attract regulatory T-cells to the tumor microenvironment, which have immunosuppressive attributes and are associated with tumor progression and worse survival (15).

While these neutrophil-related pro- and anti-tumoral mechanisms have mostly been established in the immediate tumor microenvironment, there is growing evidence that neutrophils accumulate in organs of metastasis even before the arrival of tumor cells, and form a “pre-metastatic niche” (26). The formation of the pre-metastatic niche is attributed to the release of factors supporting metastatic seeding of tumor cells. Similar to the immediate tumor microenvironment, type I IFNs were shown to inhibit the pro-tumoral differentiation of neutrophils in the pre-metastatic organs, as shown in a previous study of Wu et al., where mice deficient in interferon-alpha/beta receptor (*Ifnar1^-/-^*) developed higher metastasis loads in a mammary and lung carcinoma mouse model (9). This phenomenon depends on the release of pro-metastatic molecules, such as S100A8/9 by neutrophils infiltrating pre-metastatic lungs.

In HNC, regional metastasis to tumor-draining cervical lymph nodes occurs early during tumor progression and consequently, the lymph node status remains one of the most relevant prognostic factors for HNC patients (27). To date, mechanisms involved in the formation of a pre-metastatic niche in tumor-draining lymph nodes (TDLNs) have not been investigated. Therefore, the goal of this study was to explore the immunoregulatory activity of TDLNs-associated neutrophils prior to the development of nodal metastasis, with a special emphasis on the effects of type I IFN-signaling. A better understanding of the immunoregulatory role of neutrophils in pre-metastatic TDLNs in HNC may well open the door for novel therapeutic approaches.

## Material and Methods

### Mice

Following mice strains were used throughout the study: C57BL/6 (WT - wild type), *Ifnar1* ^-/-^, Catchup^IVM-red^ x C57BL/6, Catchup^IVM-red^ x *Ifnar1^-/-^*. In Catchup^IVM-red^ mouse model neutrophil-specific locus Ly6G is modified with a knock-in allele expressing Cre-recombinase and the fluorescent protein tdTomato. Additionally, these mice were crossed with a ROSA26 tdTomato-reporter strain to enable *in vivo* tracking of fluorescently labeled neutrophils (28). For *in vivo* luminescence imaging (IVIS^®^) a transgenic luciferase-reporter mouse model for IFN-β-production was used (IFN-β^+/Δβ-luc^ mice) (29).

For the experiments, male littermates between 7-12 weeks were used. All mice used in this study were on the C57BL/6 background. Mice were housed and bred under specific pathogen-free conditions at the animal facility of the University Hospital Essen (Essen, Germany). Animal experiments were performed according to German laws and guidelines of the Federation of European Laboratory Animal Science Associations (FELASA) and were approved by the animal ethics committee.

### Cell Line

The murine oropharyngeal carcinoma cell line MOPC (C57BL/6-derived, HPV16 E6/E7^−^) was obtained as a gift from Dr. William Chad Spanos and John H. Lee (30, 31) (Sanford Research/University of South Dakota, Sioux Falls, SD, US). During cultivation, the cell line was regularly tested for mycoplasma contamination with negative results. The cells were cultivated in a special medium (67% DMEM, 22% Hams F12 (both from Gibco by Thermo Fisher Scientific, Waltham, US), 10% Fetal Bovine Serum (FBS, PAN-Biotech, Aidenbach, Germany), 1% penicillin-streptomycin (Gibco), 0.5 µg/ml Hydrocortisone, 8.4 ng/ml Cholera Toxin, 5µg/ml Transferrin, 5 µg/ml Insulin, 1.36 ng/ml Tri-Iodo-Thyronine, 5 µg/ml E.G.F. (all from Sigma by Merck, Darmstadt, Germany) and harvested by 90% confluency. Cells were grown in a monolayer at 37°C in a humidified incubator with 5% CO_2_.

### Tumor Model

The MOPC cells were injected subcutaneously (s.c. 1 x 10^6^ in 100 µl PBS) into the flank of C57BL/6 and *Ifnar1* ^-/-^mice, tumor growth was measured by caliper every 2 to 3 days. Tumor-free animals from the same strains were used as control animals. Tumor volume was calculated as follows:


V=4/3 x π x(h x w2)/8 (h=height, w=width )

Latest on day 14 (unless stated otherwise) after tumor implantation mice were sacrificed and tissues were harvested under aseptic conditions. Blood was taken by heart puncture with a heparinized syringe and stored on ice. Tumors and inguinal TDLNs were harvested and stored in DMEMc (DMEM, 10% FBS, 1 mM sodium pyruvate and 1% penicillin-streptomycin, all from Gibco, by Thermo Fisher Scientific, Waltham, US) on ice for a maximum 30 minutes before processing.

### Preparation of a Single-Cell Suspension From LNs and Tumors

Tissue samples (tumors and TDLNs) were digested in an enzyme solution (dispase 0.2 µg/ml, collagenase A 0.2 µg/ml, DNase I 100 µg/ml, all Sigma-Aldrich/Merck, Darmstadt, Germany, in DMEMc), 1 ml per sample for 45 min at 500 rpm and 37°C. Cells were filtrated through 100 µm sterile strainers (Cell Trics, Partec, Sysmex Europe GmbH, Goerlitz, Germany), centrifuged at 300g +4°C for 5 min and the supernatant was discarded. The pellet was resuspended in 100 µl PBS containing Fc block (anti-mouse CD16/CD32 (BD, Franklin Lakes, US) and incubated for 15 min at +4°C.

### *In Vivo* Luminescence Imaging (IVIS^®^)

For *in vivo* imaging, MOPC cells were injected subcutaneously (s.c. 1 x 106 in PBS) into the flank of IFN-β+/Δβ-luc mice. On day 14 post tumor implantation mice were i.p. injected with 150 mg/kg d-luciferin (XenoLight D-luciferin, Bioluminescent Substrate, PerkinElmer, Waltham, US) in PBS, anesthetized using Isofluoran (CP-Pharma, Burgsdorf, Germany) and monitored using an IVIS^®^ 200 imaging system (Caliper LS, PerkinElmer). For IFN-β expression, photon flux was quantified at the projection of the tumor-draining inguinal lymph nodes (same side versus contralateral side) using the Living Image 4.4 software (Caliper LS, PerkinElmer) expressed in photons/s/cm2/steradian.

### Histology

On day 14 post tumor cell injection, mice were sacrificed, LNs were extracted and snap-frozen in liquid nitrogen in Tissue-Tek O.C.T. Compound (Sakura Finetek, Japan) containing 5% paraformaldehyde. Afterwards, 7-μm cryosections were prepared, fixed with -20°C cold acetone and stained with a hematoxylin-eosin, based on standard protocol. Microscopy was performed using Olympus BX51 manual upright epifluorescence microscope (Shinjuku, Japan) and tissues were analyzed manually for the presence of metastasis based on the morphology.

### Immunofluorescent Histological Staining and Fluorescent Microscopy of Lymph Nodes

Murine LNs were dissected and snap-frozen in liquid nitrogen. About 7-μm cryosections were prepared on slides and fixed with -20°C cold acetone. The sections were rehydrated in PBS, blocked with 3% goat serum in PBS and stained with anti-CD3 (clone 17A2, 3 µg/ml), anti-Ly6G (clone 1A8, 3 µg/ml), anti-CD197 (CCR7, clone 4B12, 3 µg/ml), anti-Ki-67 (clone 11F6, 3 µg/ml) and DAPI [(0,7 µg/ml), all from Biolegend, San Diego, US]. After 1.5h incubation at RT, slides were washed with PBS and ddH_2_O, dried and mounted with Neo-Mount (Merck, Burlington, US). Microscopy was performed using Zeiss AxioObserver Z1 Inverted Microscope with ApoTome Optical Sectioning equipped with filters for DAPI, FITC, Alexa Fluor 488, GFP, DsRed, and Cy3. Images were processed with ZEN Blue 2012 software and analyzed with ImageJ. Quantification of CCR7 expressing neutrophils in LNs was performed per 1 mm^2^ field of view and calculated per LN.

### Flow Cytometry Analysis

Single-cell suspensions were stained with antibodies anti-CD11b (clone M1/70, 0,20 µg/million cells) with isotype Rat IgG2b, κ (clone RTK4530, 0,20 µg/million cells), anti-Ly6G (clone 1A8, 0,20 µg/million cells), anti-CD197 (CCR7, clone 4B12, 1,0 µg/million cells) with isotype Rat IgG2a, κ (clone RTK2758, 0,20 µg/million cells), anti-CD3 (clone 17A2, 0,25 µg/million cells), anti-CD4 (clone GK1.5, 0,25 µg/million cells), anti-CD8 (clone YTS156.7.7, 0,25 µg/million cells), anti-H-2K^d^ (MHC class I, clone SF1-1.1, 0,25 µg/million cells) with isotype Mouse IgG2a, κ (clone MOPC-173, 0,25 µg/million cells), anti-CXCR4 (clone L276F12, 0,25 µg/million cells) with isotype Rat IgG2b, κ (clone RTK4530, 0,25 µg/million cells), CD80 (clone 16-10A1, 0,7 µg/million cells) with isotype Armenian Hamster IgG (clone HTK888, 0,7 µg/million cells), CD86 (clone GL-1) with isotype Rat IgG2a, κ (clone RTK2758, 0,7 µg/million cells), anti-Ki-67 (clone 11F6, 0,25 µg/million cells) with isotype (clone Rat IgG2b, κ, 0,25 µg/million cells) anti-IFN-γ (clone XMG1.2, 0,25 µg/million cells) and Flash Phalloidin ((20 units/million cells) all from Biolegend, San Diego, US)), ICAM-1 (clone KAT1, 0,25 µg/million cells) with isotype Rat IgG2b, κ (clone eBR2a, 0,25 µg/million cells), MHC-II (I-A/I-E, clone M5/114.15.2, 0,2 µg/million cells) with isotype control Rat IgG2b, κ (clone eB149/10H5, 0,20 µg/million cells), both from eBiosciences by Thermo Fisher Scientific, Waltham, US) and viability dye eFluor 780 (0,10 µg/million cells, Invitrogen by Thermo Fisher Scientific). Flow cytometry was performed using BD FACSCanto™ system and data were analyzed using BD FACSDiva™ software (BD, Franklin Lakes, US). The specific marker expression levels are shown as mean fluorescence intensity (MFI) and calculated by substruction of corresponding isotype control MFI from specific marker MFI.

### Isolation of LN Neutrophils and *In Vitro* Stimulation of T-Cells With Neutrophils

Neutrophils were isolated from LN single-cell suspensions using flow sorting (FACS Aria III Cell Sorter (BD, Franklin Lakes, US), based on the phenotype (single alive Ly6G^+^, CD11b^+^ cells) with the purity >95%. T-cells were isolated from single-cell suspension from spleen of naïve WT mice using flow sorting based on the phenotype (single alive CD3^+^ cells) with the purity >95%. Sorted neutrophils were co-incubated in DMEMc with recombinant murine IFN-λ2 (Novus Biologicals, Littleton, US) in the concentration 10 ng per 50 000 cells, during 4h at 37°C in a humidified incubator with 5% CO_2_. After sorting, T-cells were loaded with CFSE (Biolegend, San Diego, US) according to manufacturer recommendation and co-incubated with anti-CD3/28-beads (STEMCELL, Vancouver, Canada) during 72h in the absence or presence of neutrophils (WT and *Ifnar1* ^-/-^), isolated from TDLNs (50.000 neutrophils and 50.000 T-cells were used for each condition). On day 3 the cell suspension was incubated for 4h at 37°C with Brefeldin A and Monensin (for IFN-γ accumulation) followed by staining of surface and viability markers (30min at 4°C). The intracellular markers [IFN-γ and F-actin (Phalloidin)] were stained in next step, after fixation and permeabilization with the Fix/Perm Buffer Set according to the manufacturer’s instructions (all reagents from Biolegend, San Diego, US).

### *In Vitro* Multiphoton Imaging

Anesthesia and surgeries were performed in accordance with FELASA guidelines. The Catchup^IVM-red^ x C57BL/6 and Catchup^IVM-red^ x *Ifnar1^-/-^* mice were placed under ketamine/xylazine (100 µg/g/10 µg/g in 0.9% NaCl i.p.) anesthesia before surgery. During surgery and following imaging, mice were anesthetized with isoflurane (1,5% mixed with oxygen, 250 ml/min). The body temperature was kept at ∼36°C using a feedback-controlled heating pad. 30 min prior to the MP-microscopy, T lymphocytes were fluorescein-labeled by tail-vein injection of 100 μl PBS containing 10 μg FITC coupled CD3 antibody (clone 17A2, Biolegend, San Diego, US). Two-photon microscopy of the previously surgically exposed inguinal lymph node was performed using the Leica DM6000 CFS-based MP microscope with simultaneous detection *via* photomultipliers (PMTs) and hybrid reflected-light detectors (HyDs) and an HCX IRAPO L 25×/0.95-NA (numerical aperture) water-immersion objective (Leica, Wetzlar, Germany). Illumination was performed at 960 nm using a Coherent Chameleon Vision II Ti: sapphire laser. Td-Tomato-expressing neutrophils were detected with a 585/50 filter, and T-cells were detected by FITC signal with a 525/50 filter. The lymph node structures were detected by second harmonic generation (SHG) with a 460/50 filter cube. Rendering three-dimensional (3D) stacks and image processing was performed using IMARIS image visualization and analysis software (OXFORD instruments, Belfast, UK). Cells counts were performed in three different regions of interest (ROIs) of 150x150 µm per lymph node. To account cell movement over time, cell counts were performed at the beginning and at the end of the imaging time frame (15 minutes) and then averaged per ROI. Cell interactions were manually counted in ROIs over the duration of 15 minutes. Immediate cell proximity between fluorescently labeled neutrophils and T-cells in a three-dimensional plane was counted as a cell-cell interaction.

### Treatment With IFN Lambda *In Vitro*

The MOPC cells were injected subcutaneously (1 x 10^6^ s.c. in 100 µl PBS) into the flank of C57BL/6 and *Ifnar1* ^-/-^mice as described above. For therapy approach, mice were intraperitoneally injected with 8 µg of recombinant murine IFN-λ2 (Novus Biologicals, Littleton, US) in 100 µl of 0,9% NaCl solution at days 0, 2, 4, and 7 after tumor setting. Animals injected only with 100 µl of 0,9% NaCl were used as controls. Tumor growth was measured as described above. On the day 9 mice were sacrificed, the right inguinal TDLNs were removed, single-cell suspensions were prepared and flow cytometry was performed as described above.

### Treatment With IFN Lambda and *In Vitro* Multiphoton Imaging

The MOPC cells were injected subcutaneously (1 x 10^6^ s.c.) into the flank of Catchup^IVM-red^ x C57BL/6 and Catchup^IVM-red^ x *Ifnar1^-/-^* mice. The *in vivo* multiphoton imaging was performed on day 14 after tumor setting. Treatment with recombinant murine IFN-λ2 (20 µg in 100 µg PBS i.p., Novus Biologicals, Littleton, US) was performed twice: 24 hours and 1 hour before the imaging.

### Quantitative Real Time Polymerase Chain Reaction

Single cells from TDLNs were collected and stored in RNAlater (Invitrogen) RNA stabilization solution at –20C. The RNA was isolated using Qia Shredder and RNeasy Mini Kit (Qiagen, Hilden, Germany) and the cDNA was produced using the Superscript II Reverse Transcriptase Kit (Invitrogen, Thermo Fisher Scientific, Waltham, MA, US). qRT-PCR was performed at 60°C annealing temperature. As housekeeping gene, *Rps9* was used. The mRNA expression was measured using the Luna Universal qPCR Master Mix (New England BioLabs, Ipswich, MA, US). Relative gene expressions were calculated by 2^-ΔCt formulations. Primer sequences (*Rps9, Ifnb1, Tnf, Ccl19, ccl21, Cxcl1, Cxcl2*) are available upon request.

### Proteome Profiler™ Array

For analysis of cytokines profiles were used Proteome Profiler Array (Mouse XL Cytokine Array Kit, Cat.N ARY028, R&D Systems, Minneapolis, USA), based on manufacturer guidelines. In short, LN tissue (0.06 g of tissue in 0.2 ml DMEMc) was incubated for 4h at 37°C. Supernatant was collected and incubated overnight at 4°C with antibody-coated spotted membranes from the kit. After incubation, membranes were washed three times, biotin-conjugated antibody mix was added with the following incubation for one hour at room temperature. Next, the membrane was washed three times, and an HRP-conjugated antibody was added. Then, membranes were incubated in a chemiluminescence reagent that produced a light signal proportional to the amount of antibody-target analyte complexes formed. Analysis and quantification of signal intensity were done using FIJI (ImageJ) software. Signal intensities were normalized to the control spots intensities on the corresponding membranes.

### Statistical Analysis

Statistical analyses were performed using Kruskal-Wallis ANOVA with the Bonferroni correction for multiple comparisons and Mann-Whitney *U* test for two independent samples. Data are shown as median with interquartile range or as an individual values with median. P<0.05 was considered significant.

## Results

### Accumulation of Neutrophils in Tumors and Tumor-Draining Lymph Nodes Is Elevated in *Ifnar1^-/-^* Mice

**Figure 1:**
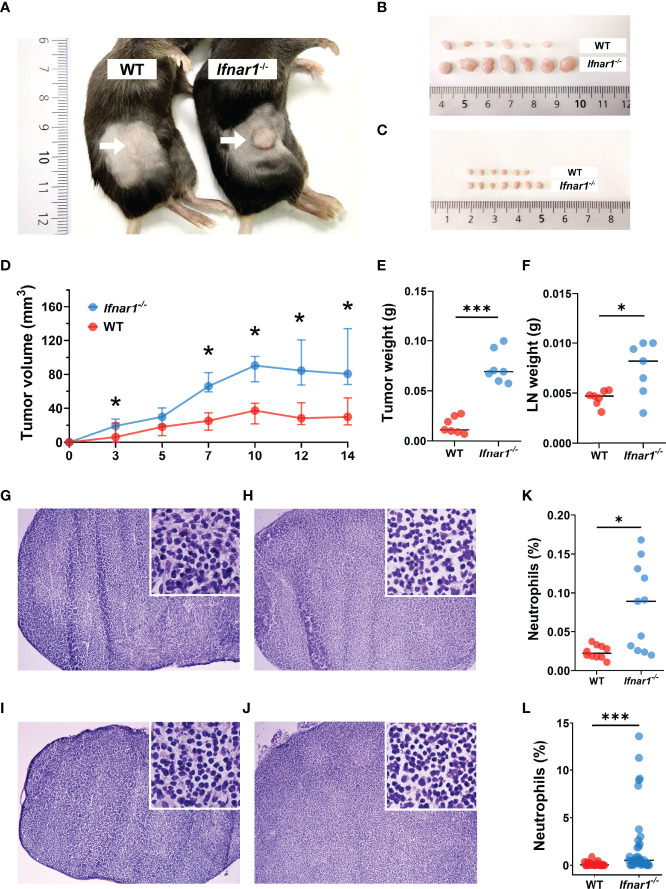
Type I IFN deficiency in mice results in accelerated tumor growth and enhanced TDLN infiltration by neutrophils. **(A, B)** Increased tumor size in *Ifnar1^-/-^* mice. Tumor cells were injected *s.c.* into the right flank of mice. Tumor volume was measured during tumor development. Mice were sacrificed on day 14 post tumor induction; tumors were isolated and analyzed. Exemplified picture of the flank tumor in mice **(A)**, comparison of tumor size between mice from one exemplified experiment are shown **(B)**. **(C)** Increased TDLNs size in *Ifnar1^-/-^* mice. Mice were sacrificed on day 14 post tumor induction; LNs were isolated and analyzed. Comparison of LN size between mice from one exemplified experiment are shown. **(D)** Accelerated tumor growth in *Ifnar1^-/-^* mice. Tumor growth was monitored 14 days. **(E)** Elevated weight of tumors in *Ifnar1^-/-^* mice. Tumors were set and monitored as described above, at day 14 tumors were removed and their weight assessed. **(F)** Higher LNs weight in *Ifnar1^-/-^* mice. **(G–J)** No metastases in TDLNs after 14 d post tumor cell injection. Hematoxylin and eosin (H&E) staining was performed on all explanted lymph nodes of tumor-bearing and tumor-free mice to confirm metastasis-free status of tumor-draining lymph nodes (TDLNs). WT tumor-free **(G)**, WT tumor-bearing **(H),** *Ifnar1^-/-^* tumor-free **(I)** and *Ifnar1^-/-^* tumor-bearing **(J)** mice. **(K, L)** The increased numbers of neutrophils in TDLNs of *Ifnar1^-/-^* tumor-bearing mice were quantified by flow cytometry **(K)** and immunofluorescent histological staining **(L).** The percentage of Ly6G^+^CD11b^+^ neutrophils from single alive cells in TDLN (presented in panel K) (gating strategy is presented in **Figure S4**). In panel **(L)** the percentage of Ly6G^+^ neutrophils was calculated from DAPI^+^ nucleated cells. Representative results are shown for (A-L). For all experiments at least three independent experiments were performed, with at least five mice per group, n=5. For comparison of two independent groups, Mann-Whitney *U* test was used. Data are shown as median with interquartile range **(D)** and individual values with median (E, F, K, L). *p < 0.05, 0.01, ***p < 0.005.

Neutrophils have been shown to control the pre-metastatic niche formation, therefore we aimed to investigate the role of TDLN-infiltrating neutrophils in this process with the focus on type I IFNs as regulators of neutrophil activity. First, we evaluated the growth of transplantable MOPC in WT and type I IFN receptor (IFNAR1) knockout (*Ifnar1* ^-/-^) mice and observed significantly elevated primary tumor growth in *Ifnar1* ^-/-^ animals (**Figures 1A, B, D, E**) compared to WT controls. Moreover, we observed significantly induced IFN-β expression in TDLNs at day 14 post tumor injection (**Figure S1**), which could be responsible for the initiation of anti-tumor adaptive immune responses in WT animals. At this time point, we observed the increased size of TDLNs in *Ifnar1* ^-/-^ animals (**Figures 1C, F**), but no visible metastases were present in TDLNs in both mouse strains (**Figure 1G–J**). In parallel, elevated neutrophil and T-cell infiltration was observed in *Ifnar1* ^-/-^ animals (**Figures 1K, L** and **S2**).

**Figure 2:**
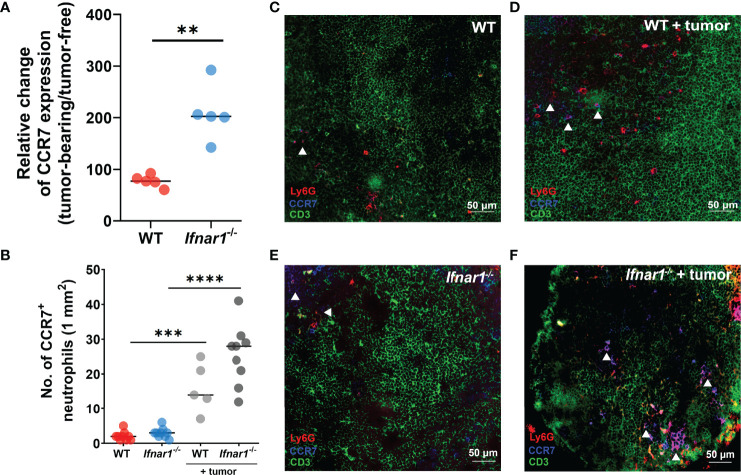
Upregulation of CCR7 expression on neutrophils leads to elevated TDLN infiltration by these cells. **(A)** Elevated expression of CCR7 receptor on neutrophils in TDLNs of tumor-bearing *Ifnar1^-/-^* mice, with relatively higher expression of CCR7 in tumor-bearing animals in comparison to tumor-free animals measured by flow cytometry **(B)** by immunofluorescent LN tissue staining. **(C–F)** Representative images of neutrophil infiltration in LNs of tumor-bearing WT **(D)** and *Ifnar1^-/-^* mice **(F)** in comparison to tumor-free animals **(C, E)**. Exemplified TDLN tissues showing immunofluorescent stained neutrophils in red, T-cells in green, CCR7 in blue, and CCR7-expressing neutrophils in magenta (indicated by white arrows); an example of single channel staining is presented on **Figure S5**. Tumor cells were injected *s.c.* into the right flank of mice. Mice were sacrificed on day 14 post-tumor induction, LNs were collected and analyzed using flow cytometry and immunofluorescent histological staining. Quantification of neutrophil infiltration in TDLNs on cryo-sections, 10 fields of view (1mm^2^) was performed. Representative results from two replicate experiments are shown, n=5 for each cohort. For comparison of two independent groups, Mann-Whitney *U* test was used. Data are shown as an individual values with median **(A, B)**. Scale bar: 50 µm. **p < 0.01, ***p < 0.005, ****p < 0.0001.

To underpin the mechanism responsible for elevated neutrophil infiltration of TDLNs in type I IFN deficiency, we analyzed the expression of CCR7 on LN neutrophils, as this receptor is suggested to be involved in the recruitment of neutrophils to T-cell zones (32). We demonstrated a significant increase of CCR7 expression on LN neutrophils in *Ifnar1^-/-^* vs WT mice in comparison to tumor-free levels (**Figure 2A**, **Figure S3**) and the elevated fraction of CCR7^+^ neutrophils in tumor-bearing *Ifnar1^-/-^* mice in comparison to tumor-free animals estimated by flow cytometry (**Figure S3**) (Neutrophils were indicated as single alive CD11b^+^Ly6G^+^ cells, gating strategy is presented on **Figure S4**; representative dot plots are presented on **Figure S3**). These findings are in line with immunofluorescent histological staining, which shows increased amounts of CCR7^+^ LN neutrophils in tumor situation, especially in *Ifnar1^-/-^* mice (**Figures 2B–F**, **S5**). In parallel with increased CCR7 expression on neutrophils, expression of CCR7 on lymphocytes in TDLN was also increased in *Ifnar1^-/-^* animals (**Figure S6**). At the same time, the expression of other molecules possibly involved in neutrophil homing into lymph nodes, such as CXCR4, CD11b (**Figures S7A, B**) and chemotaxis such as CXCL2, TNF-α, CCL21, (**Figures S7F–H**) was not significantly changed on a gene (**Figures S7C, E, G**) and protein levels (**Figures S7D, F, H**). The expression of CCL19 was changed on the gene level (**Figure S7I**), but not on protein level (**Figure S7J**).

### Characteristics of Neutrophils Infiltrating TDLNs

**Figure 3:**
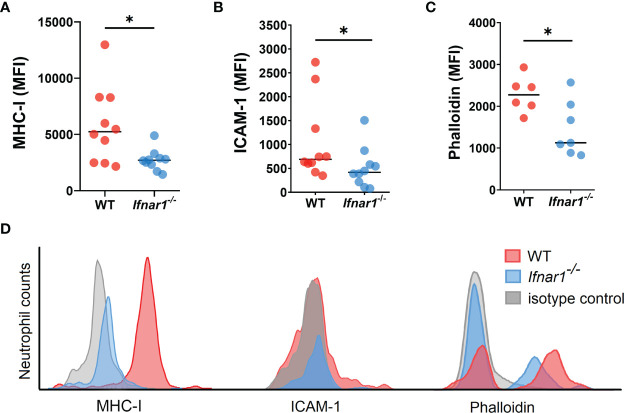
Neutrophils infiltrating TDLNs in tumor-bearing *Ifnar1^-/-^* animals show decreased expression of molecules involved in T-cell stimulation. **(A, B)** Downregulated expression of MHC-I and ICAM-1 on TDLN neutrophils from *Ifnar1^-/-^* animals. **(C)** Significantly lower levels of F-actin on TDLN neutrophils from *Ifnar1^-/-^* mice. **(D)** Representative histograms showing down-regulated expression of MHCI, ICAM-1 and phalloidin in neutrophils (single alive CD11b^+^Ly6G^+^ cells) of tumor-bearing *Ifnar1^-/-^* mice in comparison to WT mice. Tumor cells were injected *s.c.* into the right flank of mice. Mice were sacrificed on day 14 post tumor induction, LNs were collected, LN cells were stained for mentioned markers and analyzed by flow cytometry. Representative results from two replicate experiments are shown for **(A, B)**, n=10 for each cohort, and two replicate experiments are shown for **(C)**, n=6 for WT, n=7 for *Ifnar1^-/-^*. For comparison of two independent groups, Mann-Whitney *U* test was used. Data are shown as an individual values with median. *p < 0.05.

Next, we aimed to evaluate changes in the phenotype and functions of TDLN-associated neutrophils related to type I IFN deficiency. We assessed the expression of molecules involved in antigen presentation and T-cell activation on neutrophils and observed a downregulated expression of MHC-I and ICAM-1 on neutrophils derived from *Ifnar1^-/-^* animals (**Figures 3A, B, D**). At the same time, no differences in the expression of other markers associated with antigen-presenting properties of neutrophils, i.e. MHC-II, CD80 and CD86, were found (**Figures S8A–C**).

It was shown that filamentous actin (F-actin) polarization at the immune synapse of antigen-presenting cells maintains synapse stability necessary to induce T-cell activation (33). We performed the phalloidin staining of isolated LN neutrophils to check cytoskeleton organization with flow cytometry, as microscopical evaluation of F-actin in neutrophils is impeded due to the size and complex shape of the nucleus (34). We detected a significantly lower F-actin levels in *Ifnar1^-/-^* as compared to WT (**Figures 3C, D**), suggesting decreased F-actin polarization and therefore impaired immunological synapse stability in IFN I deficiency as a possible reason for impaired interaction between neutrophils and T-cells in TDLNs.

Since MHC-I and ICAM-1 are known to be involved in the formation of immunological synapse between APCs and T-cells, we aimed to visualize the interactions between neutrophils and T-cells in regional LNs of tumor-bearing animals.

### IFN I Deficiency as a Reason for Impaired Interaction Between Neutrophils and T-Cells in TDLNs

**Figure 4:**
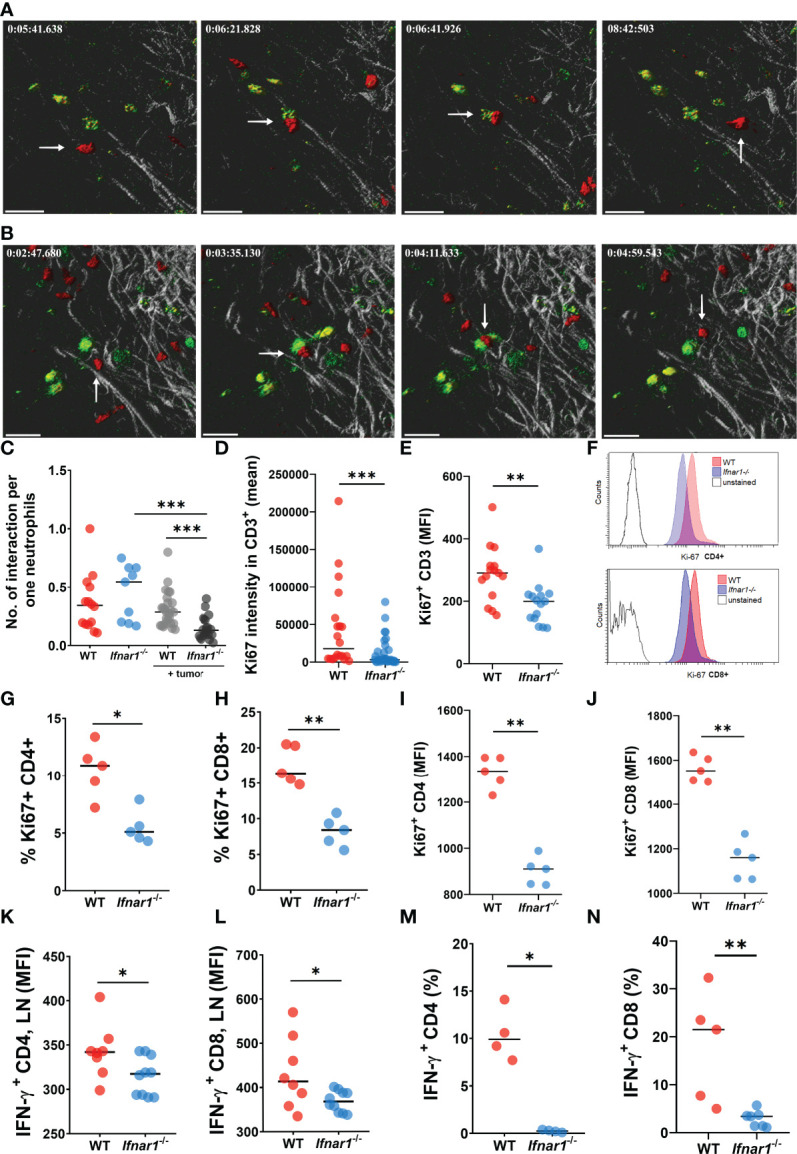
Impaired interactions between neutrophils and T-cells in TDLNs. **(A, B)** Tumor decreases the number of interactions between neutrophils and T-cells in LNs of tumor-bearing WT **(A)** and *Ifnar1^-/-^* **(B)** mice. Exemplified pictures showing TDLN neutrophils in red (tdTomato), T-cells in green (FITC), and LNs tissue in grey (autofluorescence). Corresponding movies available in supplements. Scale bar 30 µm. The white arrows indicate the neutrophil that interacts with T-cell. **(C)** Growing primary tumor impairs interactions between neutrophils and T-cells in TDLNs of tumor-bearing animals, especially in *Ifnar1^-/-^* mice, in comparison to tumor-free animals (interactions were estimated during 15 minutes and normalized per one neutrophil, in an *in vivo* system)**. (D–J)** Significantly reduced T-cell activation in TDLNs in *Ifnar1^-/-^* mice, estimated by Ki-67 expression on TDLN cryo-sections **(D)** and flow cytometry for all T Lymphocytes **(E)** and for both CD8 and CD4 subpopulations **(G–J)**. **(F)** Representative histograms showing down-regulated expression of Ki-67 in CD4 and CD8 cells of tumor-bearing *Ifnar1^-/-^* mice in comparison to WT mice. **(K, L)** Impaired IFN-ɣ expression in CD4 **(K)** and CD8 (L) cells in TDLNs of *Ifnar1^-/-^*. **(M, N)** Suppressed T-cell activation after co-incubation with *Ifnar1^-/-^* TDLN neutrophils visualized by IFN-γ production in both CD4 **(M)** and CD8 **(N)** cell populations, in an *in vitro* setting. For **(A–C)** tumor cells were injected *s.c.* into the flank of mice. Two-photon microscopy of the previously surgically exposed inguinal TDLNs was performed on day 14. Cell interactions were manually counted in ROIs over the duration of 15 minutes. Representative results from six replicate experiments are shown for **(A, B)**, n=5 for WT animals, n=8 for WT tumor-bearing animals, n=5 for *Ifnar1^-/-^* animals, n=8 for *Ifnar1^-/-^* tumor-bearing animals. For **(D–J, K, L)** tumor cells were injected *s.c.* into the flank of mice. Mice were sacrificed on day 14 post tumor induction, LNs were collected, immunofluorescent histological staining (quantification of CD3^+^ and CD3^+^Ki67^+^ cells, 10 fields of view 1mm^2^) **(D)** and flow cytometry (Ki67 expression on single alive CD3^+^, CD3^+^CD4^+^ and CD3^+^CD8^+^ cells) **(E–G)** were performed. Representative results are shown for thee experiment for **(D)**, n=10 for each cohort and for three experiments for **(E)**, n=5 for each cohort and three replicate experiments. Representative results are shown for two replicate experiments for **(G–J)**, n=5 for each cohort. For **(M, N)** tumor cells were injected *s.c.* into the flank of mice. Mice were sacrificed on day 14 post tumor induction, LNs were collected and neutrophils (single alive Ly6G^+^ CD11b^+^ cells) were isolated using flow sorting. T-cells (single alive CD3^+^ cells) were isolated from the spleens of naïve WT mice using flow sorting and co-incubated with neutrophils. On day 3 the intracellular expression of IFN-ɣ in single alive CD4^+^ and CD8^+^ cells was estimated by flow cytometry. Representative results are shown for two experiment for **(M)**, n=4 for each cohort and two replicate experiments for **(N)**, n=5 for WT, n=7 for *Ifnar1^-/-^*. For comparison of two independent groups, Mann-Whitney *U* test was used. Data are shown as an individual values with median. *p < 0.05, **p < 0.01, ***p < 0.005.

The Catchup^IVM-red^ mice were used for imaging of neutrophils and their cell/cell interactions in tumor-draining inguinal LNs *via* two-photon intravital microscopy. To assess the interaction of neutrophils with T Lymphocytes in such LNs *in vivo*, T Lymphocytes were labeled with FITC-coupled CD3 antibody. We observed that tumor injection led to a significant downregulation of neutrophil/T-cell interactions in TDLNs of *Ifnar1^-/-^* mice, but not in WT mice (**Figures 4A–C**) (**Supplementary Movies 1**, **2**). Naïve non-tumor-bearing WT and *Ifnar1^-/-^* mice were used as a control. We did not observe any differences in interaction numbers between control groups (**Figure 4C**). Of note, to avoid artifacts due to different neutrophil numbers in LN, the number of neutrophil/T-cell interactions was recalculated per one neutrophil.

### Neutrophils Deficient in Type I IFN Signaling Show Decreased Capacity to Activate T-Cells

The observed decrease of neutrophil interactions with T-cells in *Ifnar1^-/-^* mice could lead to the impaired activation and proliferation of effector T Lymphocytes. Indeed, we observed significantly reduced activation of T-cells responses in TDLN tissue and isolated cells of *Ifnar1^-/-^* mice, estimated by Ki-67 expression (**Figures 4D–J** and **S9**). In line with that, lower IFN-γ expression in CD4^+^ and CD8^+^ T-cells in TDLNs was observed (**Figures 4K, L**)

To prove the role of neutrophils in activation of T-cells *in vitro*, we co-incubated isolated WT vs. *Ifnar1^-/-^* TDLN neutrophils with WT naïve T-cells. Indeed, we observed significantly suppressed T- cell activation after co-incubation with *Ifnar1^-/-^* neutrophils, as visualized by reduced IFN-γ production by both CD4^+^ and CD8^+^ T Lymphocytes (**Figures 4M, N**).

Altogether, these results suggest that deficiency in type I IFNs might be responsible for impaired neutrophil/T-cell interactions, resulting in impaired activation of anti-tumor immune responses. This in turn could be responsible for the observed accelerated tumor growth in IFNAR1-deficient mice.

### Activation of IFN Signaling Downstream of Non-functional IFNAR1 Rescues T-Cell Stimulatory Capacity of Neutrophils in *Ifnar1^-/-^* Mice *In Vitro* and *In Vivo*

Therapeutic strategy aiming to restore type I IFN signaling in neutrophils could enhance their anti-tumoral properties (35). Among others, type III interferons (interferon lambda, IFN-λ) are the most functionally similar to type I IFNs and share downstream signaling pathway, although signaling through distinct receptors (36). Therefore, we aimed to evaluate whether IFNAR1 deficiency could be rescued by the activation of IFN signaling downstream of the inactive receptor, using IFN-λ, hereby potentially activating immunostimulatory properties of *Ifnar1^-/-^* TDLN neutrophils.

**Figure 5:**
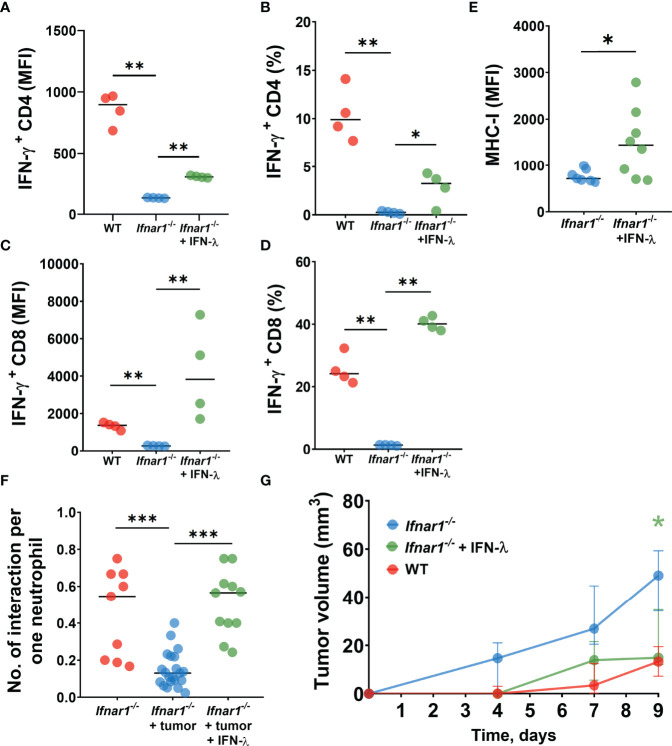
IFN-λ treatment increases neutrophil/T-cell interactions, activates T-cell and reduces tumor growth. **(A–D)** LN neutrophils from *Ifnar1^-/-^* tumor-bearing mice after *in vitro* treatment with IFN-λ significantly improved their capacity to stimulate IFN-ɣ production by T-cells. **(E)** Upregulation of MHC-I expression on TDLN- infiltrating *Ifnar1^-/-^* neutrophils after *in vivo* treatment with IFN-λ. **(F)** Enhanced numbers of interactions between neutrophils and T-cells in TDLNs in *in vivo* IFN-λ treated *Ifnar1^-/-^* mice. **(G)** Significantly decreased tumor growth in *in vivo* IFN-λ treated *Ifnar1^-/-^* mice. For **(A–D)** tumor cells were injected *s.c.* into the flank of mice. Mice were sacrificed on day 14 post tumor induction, LNs were collected, neutrophils (single alive Ly6G^+^CD11b^+^ cells) were isolated using flow sorting and treated with IFN-λ. T-cells (single alive CD3^+^ cells) were isolated from the spleens of naïve WT mice using flow sorting and co-incubated with pre-treated neutrophils. On day 3 the intracellular expression of IFN-ɣ in single alive CD4^+^ and CD8^+^ cells was estimated by flow cytometry. Representative results from one experiment are shown for **(A–D)**, n=15 for WT animals, n=14 for *Ifnar1^-/-^* animals. Isolated LN neutrophils were pooled and divided in three groups. For **(E, F)** tumor cells were injected *s.c.* into the flank of mice and treatment with IFN-λ was applied at days 13 and 14 (24 hours and 1 hour before the imaging procedure). Mice were sacrificed on day 14 post tumor induction, LN were collected and MHC-I expression on neutrophils (single alive Ly6G^+^CD11b^+^ cells) was estimated by flow cytometry **(E)**. Representative results from two replicate experiment are shown for **(E)**, n=6 for WT animals, n=7 for *Ifnar1^-/-^* animals. Two-photon microscopy of the previously surgically exposed inguinal lymph node was performed on day 14 and cell interactions were manually counted in ROIs over the duration of 15 minutes **(F)**. Representative results from four replicate experiments are shown for **(F)**, n=5 *Ifnar1^-/-^* animals, n=8 *Ifnar1^-/-^* tumor-bearing animals, n=4 IFN-λ treated *Ifnar1^-/-^* tumor-bearing animals. For **(G)** tumor cells were injected *s.c.* into the flank of mice. Treatment with IFN-λ was applied during the tumor progression and tumor volume was measured. Mice were sacrificed on day 9 post-tumor induction. Representative results from two replicate experiment are shown for **(G)**, n=5 for each cohort. For comparison of two independent groups, Mann-Whitney *U* test was used, and for comparison of multiple independent groups Kruskal-Wallis (ANOVA) was used. Data are shown as median with interquartile range **(G)** and individual values with median **(A–F)**. *p < 0.05, **p < 0.01, ***p < 0.005.

We first isolated *Ifnar1^-/-^* TDLN neutrophils and treated them *in vitro* with IFN-λ to evaluate their capacity to stimulate T-cells. Indeed, we observed elevated T-cell activity indicated by their IFN-γ production after co-incubation with such treated neutrophils, as compared to untreated *Ifnar1^-/-^* cells (**Figures 5A–D**). Moreover, treatment with IFN-λ increased MHC-I expression by TDLN neutrophils *ex vivo* (**Figure 5E**).

Next, we assessed the capacity of IFN-λ to rescue cell stimulatory properties of IFNAR1-deficient neutrophils *in vivo*. To this end, we treated *Ifnar1^-/-^* tumor-bearing mice with IFN-λ and monitored neutrophil/T-cell interactions using the *in vivo* multiphoton imaging, as described above. In agreement with our *in vitro* data, we observed elevated counts of interactions between neutrophils and T-cells in TDLNs of IFN-λ treated mice, compared to untreated animals (**Figure 5F**).

Finally, to prove the therapeutical effect of IFN-λ on anti-tumor immune responses and tumor growth in IFNAR1-deficient animals *in vivo*, we set tumors into mice and treated animals with IFN-λ as described in methods. Tumor growth was monitored and mice sacrificed at day 9. Indeed, *Ifnar1^-/-^* mice treated with IFN-λ show significantly decreased tumor growth, as compared to untreated *Ifnar1^-/-^* mice (**Figure 5G**).

Collectively, these data suggest that inactive IFNAR1 on neutrophils leads to the prominent defect in the neutrophil-mediated activation of anti-tumor T-cell responses in TDLNs. IFNAR1-independent rescue of type I IFN signaling, using IFN-λ, improves the immunostimulatory capacity of neutrophils in TDLNs and contributes to the suppression of tumor growth.

## Discussion

In this study, we performed an in-depth *in vivo* and *ex vivo* analysis of the immunogenic microenvironment of tumor-draining lymph nodes (TDLN) in a transplantable murine head and neck model. To avoid systemic immunosuppressive effects and systemic spreading of tumor antigen-specific T-cells during tumor development (37) we used early-stage cancer model. At this time point, no metastases are observed in LNs.

Overall, our results suggest that neutrophil-mediated regulation of anti-cancer immune responses in lymph nodes is modulated, at least in part, by type I IFN signaling. Deactivation of type I IFN receptor (IFNAR1) on neutrophils leads to a significant reduction of neutrophil/T-cell interactions accompanied by the decreased activity and proliferation of the T cells, and elevated tumor growth in such mice. *Ex vivo* analyses confirmed the reduced capacity of such type I IFN-deficient neutrophils to stimulate T-cells, as measured by a significantly suppressed IFN-γ production by both CD4^+^ and CD8^+^ cells.

Previously, we could already show for different tumor entities that the deficiency in type I IFN signaling leads to the elevated tumor growth in *Ifnar1^-/-^* mice and changed tumorigenic properties of tumor-associated neutrophils (9). Type I IFN signaling in the immediate tumor microenvironment has also been shown to stimulate anti-cancer immunosurveillance as well as to reduce the metastatic dissemination of carcinomas, which is associated with the progressive tumor growth (22, 23). These findings stem from animal models as well as human tissue analysis for different types of solid cancers. In mice, the metastatic spread of mammary or lung carcinoma has been shown to be accelerated in *Ifnar1^-/-^* mice (9). Similar effects were demonstrated in human melanoma cells, where an impaired production of type I IFN by cancer cells was associated with bone metastasis formation (38). In colorectal cancer, IFNAR1 was shown to be actively degraded in the tumor tissue by phosphorylation-dependent ubiquitination, which was supported by VEGF (39). This led to the impairment of type I IFN signaling and resulted in the immunosuppression that supported tumor growth (40). Our primary tumor analyses suggest the existence of similar mechanisms in head and neck cancer.

In the light of their prognostic importance for HNC, but also due to their relevance for the initiation of anti-tumor immune responses, we thereafter focused on the analysis of pre-metastatic tumor-draining lymph nodes. In particular, we chose to analyze lymph nodes prior to the formation of metastases, which represent an intriguing therapeutic target in HNC patients. Our analyses revealed that TDLN-associated neutrophil counts in tumor-bearing mice were overall significantly elevated, compared to tumor-free animals. Notably, significantly more neutrophils accumulated in TDLNs of tumor-bearing *Ifnar1^-/-^* mice compared to tumor-bearing WT controls. In light of strongly elevated primary tumor growth in *Ifnar1^-/-^* mice this phenomenon suggests pro-tumoral bias of such LN neutrophils. As shown for other tumor entities, IFNAR1 deficiency is a well-documented driver of pro-tumoral differentiation of neutrophils (8, 41, 42), as well as neutrophil migration to organs of metastasis, even prior to metastasis formation (9). To further illuminate the mechanism of neutrophil recruitment, we performed analyses of CCR7 expression on LN neutrophils and observed significant upregulation of this receptor on *Ifnar1^-/-^* cells in tumor-bearing animals as compared to tumor-free mice. The role of CCR7 as a receptor facilitating migration of neutrophils to lymph nodes has been previously suggested (32). In parallel with increased CCR7 expression on *Ifnar1^-/-^* neutrophils, lymphocytes from *Ifnar1^-/-^* TDLN were also characterized by the elevated CCR7 expression. Such phenomenon can be additionally responsible for increased migration of lymphocytes to TDLN and elevated size of TDLN that is observed in *Ifnar1^-/-^ mice*. In agreement with our data, activation *via* IFNAR1 was reported to regulate CCR7 on different cell types (43)

In recent years, the immunoregulatory functions of neutrophils in the tumor microenvironment have been described. Neutrophils appear to interact with T-cells in multiple ways, including the presentation of antigens for T-cell activation, as shown in lung cancer model (16). Recruitment of T-cells by neutrophils is known to be facilitated by cytokines, such as TNF-α, Cathepsin G, and neutrophil elastase (15). Our *in vivo* analyses allowed a detailed estimation of spatiotemporal neutrophil/T-cell interactions in TDLNs. We could demonstrate significantly increased counts of neutrophil/T-cell interactions in tumor-bearing mice, compared to non-tumor-bearing controls. However, tumor-bearing *Ifnar1^-/-^* mice show significantly less T-cell interactions per neutrophil in comparison to WT mice. The expression of ICAM-1 expression by neutrophils could be one of the mechanisms involved in this process (18). ICAM-1 is a co-stimulatory ligand expressed on APCs that was demonstrated to stabilize cell-cell interactions and to facilitate antigen presentation to lymphocytes (44, 45). Reduced expression of MHC-I of the neutrophil surface could also lead to impaired contacts with T-cells (46). The actin cytoskeleton plays also a crucial role in the formation and maintenance of the immunological synapse between antigen-presenting cell and T-cell (47). Interestingly, we observed a reduction of F-actin signal in *Ifnar1^-/-^* neutrophils, which could indicate the decreased capacity of these cells to form immunological synapses with T-cells.

Lower counts of neutrophil/T-cell interactions in *Ifnar1^-/-^* mice were accompanied by the reduced proliferation of T-cells in LN of such mice. Moreover, stimulation of WT T-cells with *Ifnar1^-/-^* neutrophils resulted in decreased activity and proliferation of these T-cells, as evaluated by down-regulated Ki67-staining and decreased IFN-γ expression. Altogether, these results suggest that IFN-deficiency does not only reduce the number of interactions between neutrophils and T-cells, but in addition, it leads to less efficient activation of T-cells. These findings are in agreement with previous data showing the supporting role of IFNs in the activity and antigen presentation capacity of APCs, including monocytes and dendritic cells (DCs) (48, 49).

With regard to a potential therapeutic approach, we attempted a rescue experiment using the systemic application of IFN-λ to *Ifnar1^-/-^* mice. Interferon IFN-λ, the member of type III IFNs, signals *via* distinct receptor complex, but activates common Janus kinase (JAK) and signal transducer and activator of transcription (STAT) pathways, similar to IFN-α/β. Moreover, type III IFNs share many biological activities with type I IFNs (36). Beside direct effects on cancer cells and T lymphocytes (50), recent studies demonstrated the capacity of IFN-λ to promote NK-cell mediated innate immunity in solid cancer models (51, 52). In addition, IFN-λ has recently been suggested to play a role in neutrophil stimulation during inflammatory processes (53). Importantly, we observed that IFN-λ treatment of tumor-bearing *Ifnar1^-/-^* mice led to a significant increase of neutrophil/T-cell interactions in TDLNs of these mice, to the levels observed in WT mice. This was accompanied by significantly reduced tumor growth in such IFN-λ−treated mice. Treatment of isolated LN-associated neutrophils with IFN-λ *in vitro* rescued the capacity of these cells to activate T-cells and possibly to stimulate T-cell proliferation. Such therapeutic intervention might be useful not only to suppress primary tumor growth, but also to prevent the formation of metastases, which would be of particular interest in the treatment of HNC (27). Nevertheless, more research is needed to evaluate this possibility. While potential adverse effects of a systemic administration of type I IFNs have been well documented (54), targeted approaches may be more promising and have been successfully applied in animal models (55). For HNC applications, recently reported synergistic effects of type I IFN and checkpoint inhibitors, which have been approved for clinical use in the past years, may be of particular value (56).

Among other mechanisms of tumor-driven immunosuppression, the downregulation of IFNAR1 plays a special role, decreasing the effectiveness of anti-tumor innate and adaptive immune responses, which enables tumor growth and permits the formation of pre-metastatic niches, minimizing the efficiency of anti-cancer type I IFN therapy. Optimization of therapeutical strategies rescuing the type I IFN signaling, e.g. with IFN-λ, improves the immunostimulatory properties of neutrophils and thus restores the anti-cancer activity of lymphocytes, providing an attractive therapeutical option for cancer treatment.

## Data Availability Statement

The raw data supporting the conclusions of this article will be made available by the authors, without undue reservation.

## Ethics Statement

The animal study was reviewed and approved by Federation of European Laboratory Animal Science Associations (FELASA).

## Author Contributions

Study concept: JJ; Study design: TH, MD, EP, SB, and JJ; Data acquisition: TH, MD, SB, EP, ES, IS, IO, AS; Quality control of data and algorithms: TH, MD, SB, EP, ES and JJ; Data analysis and interpretation: MD, TH, EP, SB, ES and JJ; Statistical analysis: MD, TH, EP, SB; Manuscript preparation: MD, TH, EP, SB, ES and JJ; Manuscript editing: TH, MD, EP, ES, SB, IS, FD, IO, KS, AH, SLi, MG, SLa and JJ, Resources: SLa and JJ. All authors contributed to the article and approved the submitted version.

## Funding

This work was supported by Deutsche Forschungsgemeinschaft (DFG/JA 2461/2-1 and DFG/JA 2461/5-1) and Deutsche Krebshilfe (No. 70113112). EP was supported by the IFORES PEP-BIOME excellence program of University of Duisburg-Essen.

## Conflict of Interest

The authors declare that the research was conducted in the absence of any commercial or financial relationships that could be construed as a potential conflict of interest.

## Publisher’s Note

All claims expressed in this article are solely those of the authors and do not necessarily represent those of their affiliated organizations, or those of the publisher, the editors and the reviewers. Any product that may be evaluated in this article, or claim that may be made by its manufacturer, is not guaranteed or endorsed by the publisher.
