# Supplementary material for: IFNAR1 Deficiency Impairs Immunostimulatory Properties of Neutrophils in Tumor-Draining Lymph Nodes
Source: Front Immunol. 2022 Jun 27;13:878959. doi: 10.3389/fimmu.2022.878959 (PMC9271705; doi:10.3389/fimmu.2022.878959)
Supplement: Supplementary file 1 [file DataSheet_1.docx]

Supplementary Material

IFNAR1 deficiency impairs immunostimulatory properties of neutrophils in tumor-draining lymph nodes

Timon Hussain^1†^, Maksim Domnich^1†^, Sharareh Bordbari^1^, Ekaterina Pylaeva^1^, Elena Siakaeva^1^, Ilona Spyra^1^, Irem Ozel^1^, Freya Droege^1^, Anthony Squire^2^, Stefan Lienenklaus^3^, Kathrin Sutter^4^, Anja Hasenberg^2^, Matthias Gunzer^2,5^, Stephan Lang^1,6^, Jadwiga Jablonska^1,6^*

***Correspondence:**
**J. Jablonska, Translational Oncology, Dept. of Otorhinolaryngology, University Hospital, University of Duisburg-Essen, Hufelandstraße 55, 45147 Essen, Germany. Phone: +49(201)7233190; E-mail:** [**jadwiga.jablonska@uk-essen.de**](mailto:jadwiga.jablonska@uk-essen.de)

**Figures**


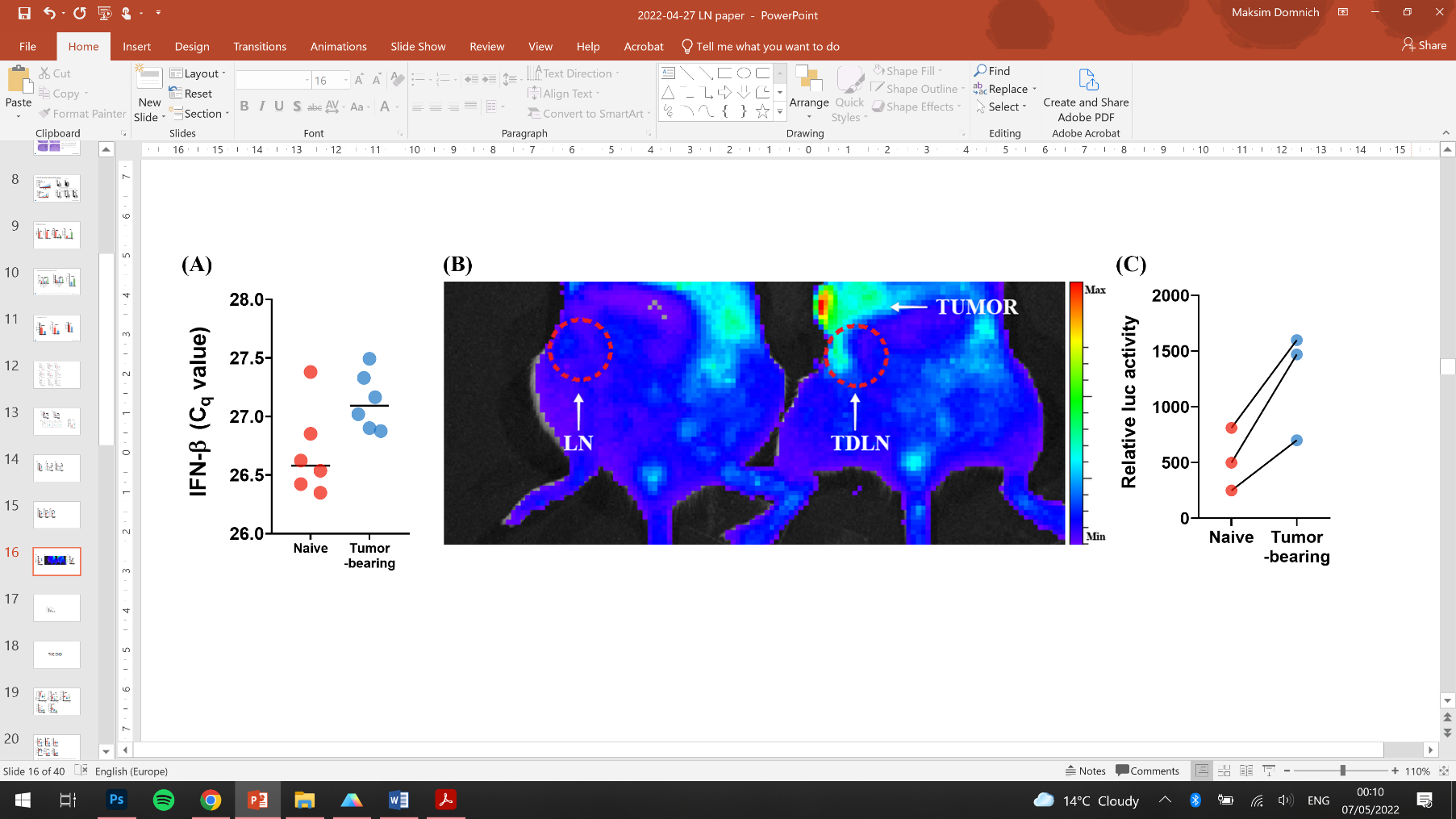


**Figure S1.** **Upregulated production of IFN-β in the tumor tissue and TDLNs during cancer progression. (A)** Evaluated expression of IFN-β in tumor-bearing naïve mice. (B, C) Bioluminescence image of luciferase reporter mice (IFNβ^+^/Δβ-luc); images taken on day 14 post MOPC tumor injection. The white arrow indicates healthy LN (red cycle on the left mouse) and TDLN (red cycle on the right mouse) and tumor with high expression of IFN-β.


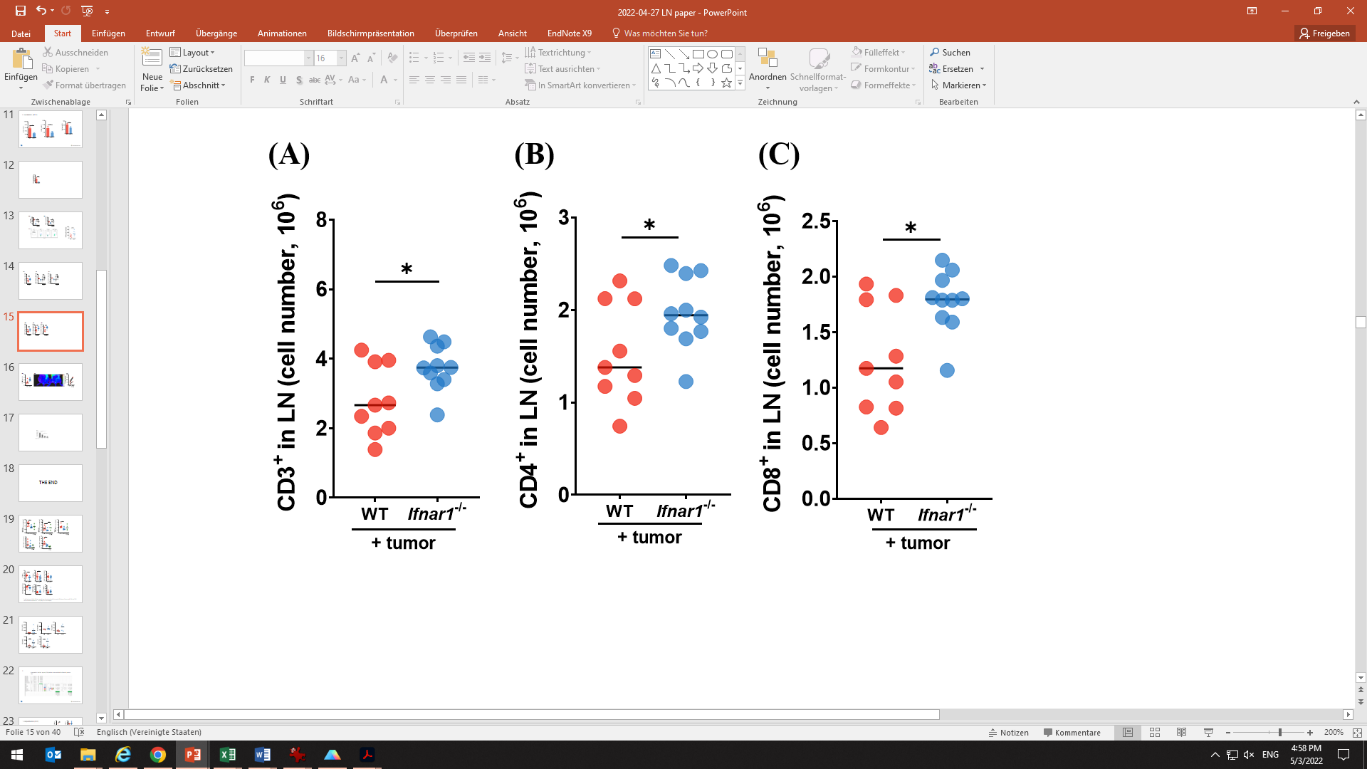


**Figure S2. Elevated amounts of T-cells in TDLNs of *Ifnar1^-/-^* mice.** (**A-C**) Altered number of T-cells in TDLNs of *Ifnar1*^-/-^ mice. The numbers of CD3^+^ (A), CD4^+^ (B) and CD8^+^ (C) cells were assessed in TDLNs of *Ifnar1*^-/-^ versus to WT animals. MOPC cells were injected subcutaneously (1 x 10^6^ cells in 100 µl PBS) into the flank of WT (C57BL/6) and *Ifnar1^-/-^* mice. On day 14 mice were sacrificed, TDLNs were isolated, single-cell suspension was prepared, stained and analyzed by flow cytometry. The number of T-cells (single alive CD11b^+^ Ly6G^+^ cells) was estimated per TDLN.


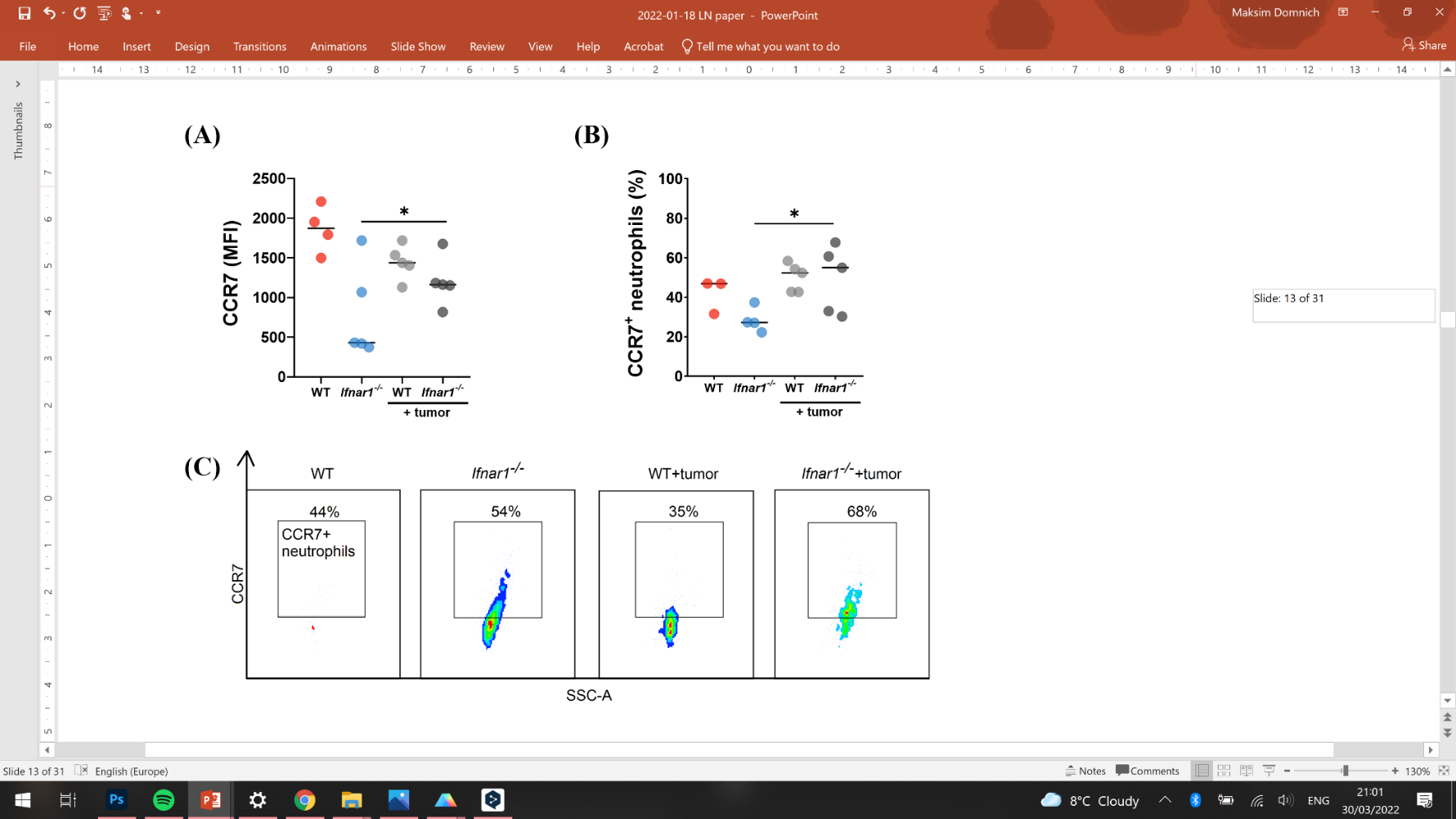


**Figure S3. The CCR7 expression and the fraction of CCR7^+^ LN-infiltrating neutrophils noticeably increase in tumor-bearing condition in *Ifnar1^-/-^* mice.** **(A)** Increased CCR7 expression on LN neutrophils in tumor-bearing *Ifnar1^-/-^* mice in comparison to tumor-free mice. **(B)** Elevated fraction of CCR7^+^ LN neutrophils in tumor-bearing *Ifnar1^-/-^* mice. **(C)** Representative dot plots showing difference of CCR7^+^ LN neutrophils in tumor-free and tumor-bearing WT and *Ifnar1^-/-^* mice. The MOPC cells were injected subcutaneously (1 x 10^6^ cells in 100 µl PBS) into the flank of WT (C57BL/6) and *Ifnar1^-/-^* mice. On day 14 mice were sacrificed, tumor-draining inguinal LNs were dissected. The inguinal LNs of tumor-free mice (both strains) were used as a control. Single-cell suspensions from LNs were prepared, stained and analyzed by flow cytometry. The CCR7 expression on neutrophils (single alive CD11b^+^ Ly6G^+^ cells) and the percentage of CCR7^+^ neutrophils from all neutrophils was estimated. Data are shown as an individual values and median, *p < 0.05.


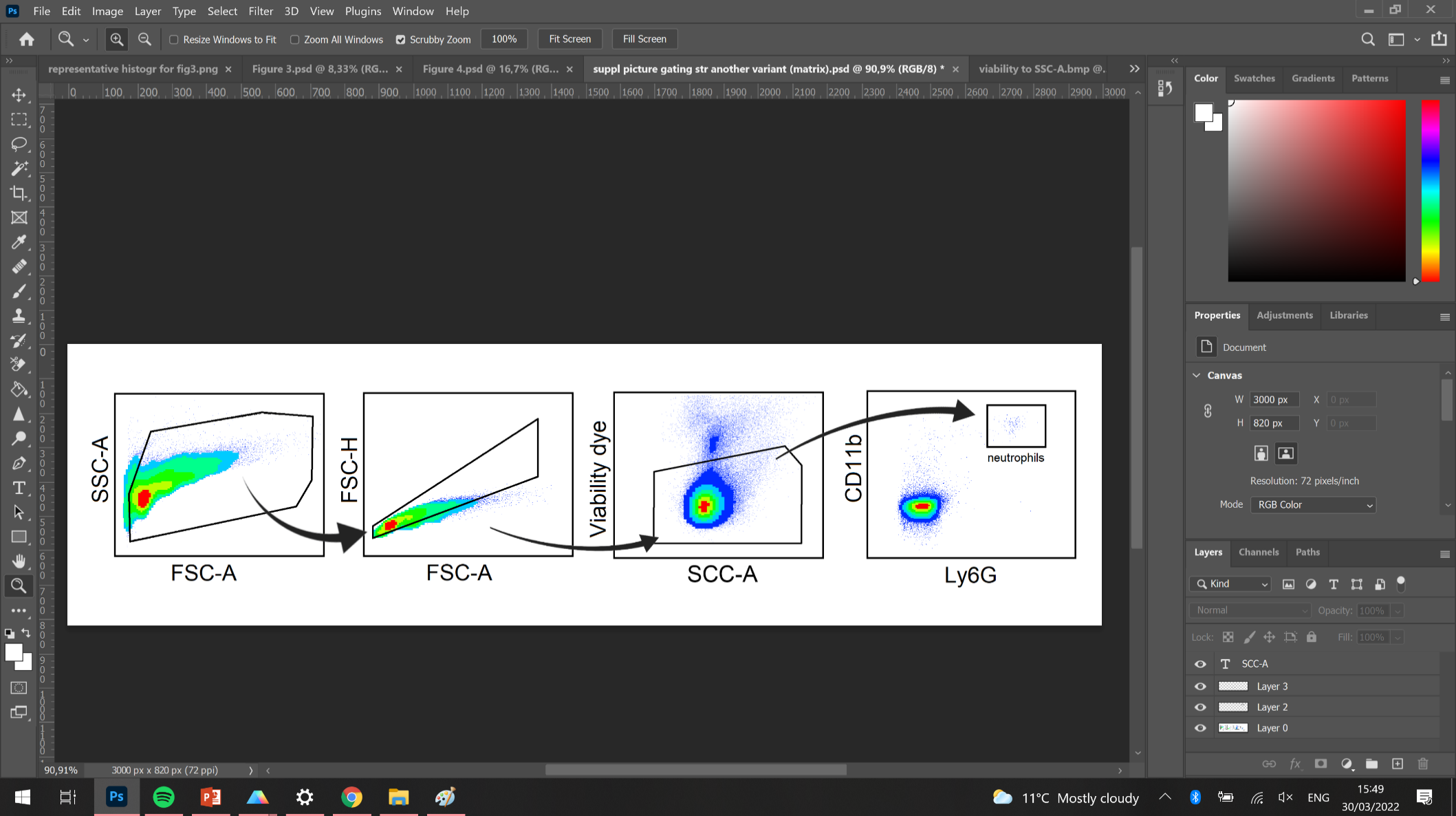


**Figure S4.** **Gating strategy for TDLN-infiltrating neutrophils (single viable CD11b^+^ Ly6G^+^ cells).** The MOPC cells were injected subcutaneously (1 x 10^6^ cells in 100 µl PBS) into the flank of WT (C57BL/6). On day 14 mice were sacrificed, tumor-draining LNs were dissected, single-cell suspension was prepared, stained for mentioned markers and analyzed according the showed flow cytometry analysis.


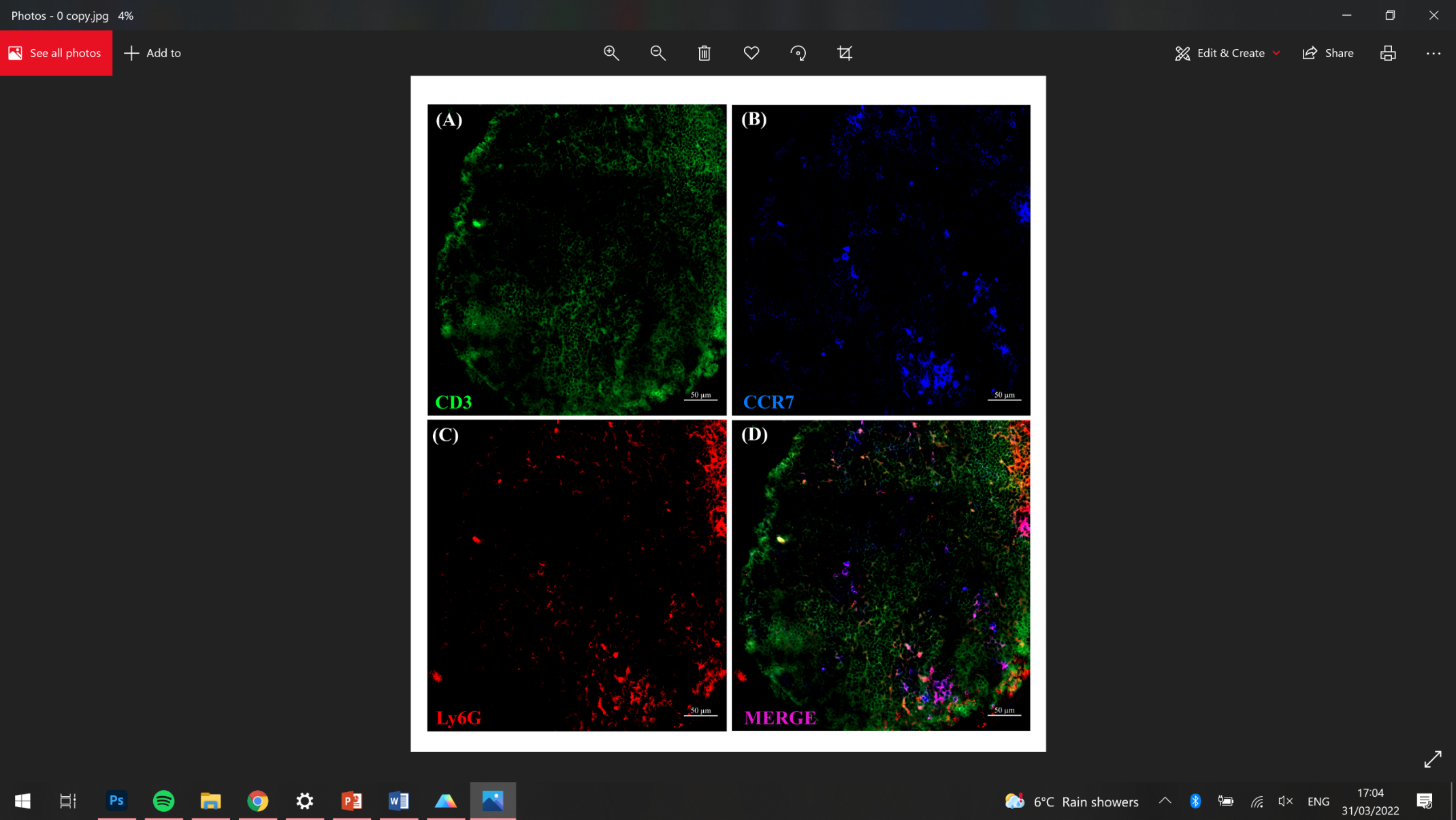


**Figure S5.** **Representative image of neutrophil distribution in LNs of tumor-bearing *Ifnar1^-/-^* mouse** (see Figure 2F). Immunofluorescent staining of LN tissue with (**A**) T-cells in green, (**B**) CCR7 in blue, (**C**) neutrophils in red and (**D**) CCR7-expressing neutrophils in magenta.


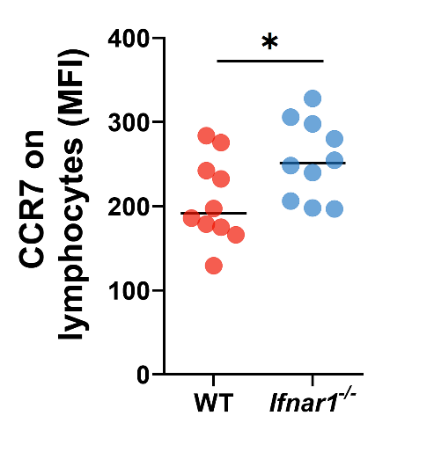


**Figure S6. Elevated expression of CCR7 on** **lymphocytes from TDLNs.** Significant increase in tumor-bearing condition in *Ifnar1^-/-^* mice. On day 14 post tumor implantation mice were sacrificed, tumor-draining LNs were dissected, single-cell suspension was prepared, stained for CCR7 and analyzed on lymphocytes by flow cytometry.


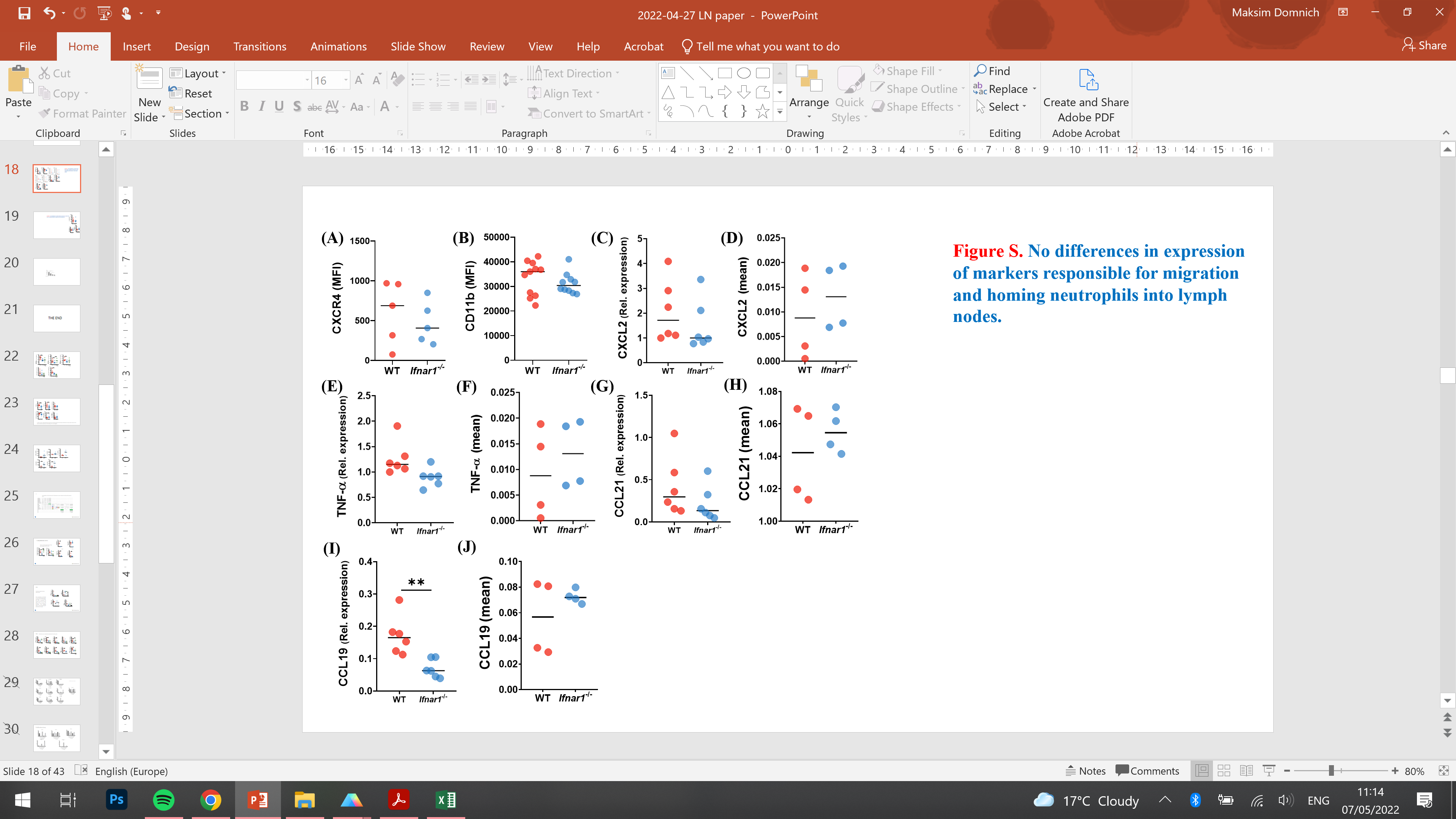


**Figure S7.** **No differences in expression of markers responsible for chemotaxis, migration and homing of neutrophils into lymph nodes, except of CCL19.** (**A-H**) The lack of significant differences in expression of chemotaxis and migratory markers. (**A**) CXCR4, (**B**) CD11b, (**C, D)** CXCL2, (**E, F) TNF-**α, (**G, H**) CCL21. (**I, J**) Expression of CCL19 was changed on the gene level (**I**) but not on protein level (**J**). The MOPC cells were injected subcutaneously (1 x 10^6^ cells in 100 µl PBS) into the flank of WT (C57BL/6). On day 14 post tumor implantation mice were sacrificed, tumor-draining LNs were dissected and prepared for analysis by flow cytometry (**A, B**), qPCR (**C, E**, **G, I**) and proteome profiler assay (**D, F, H, J**). Data are shown as an individual values and median.


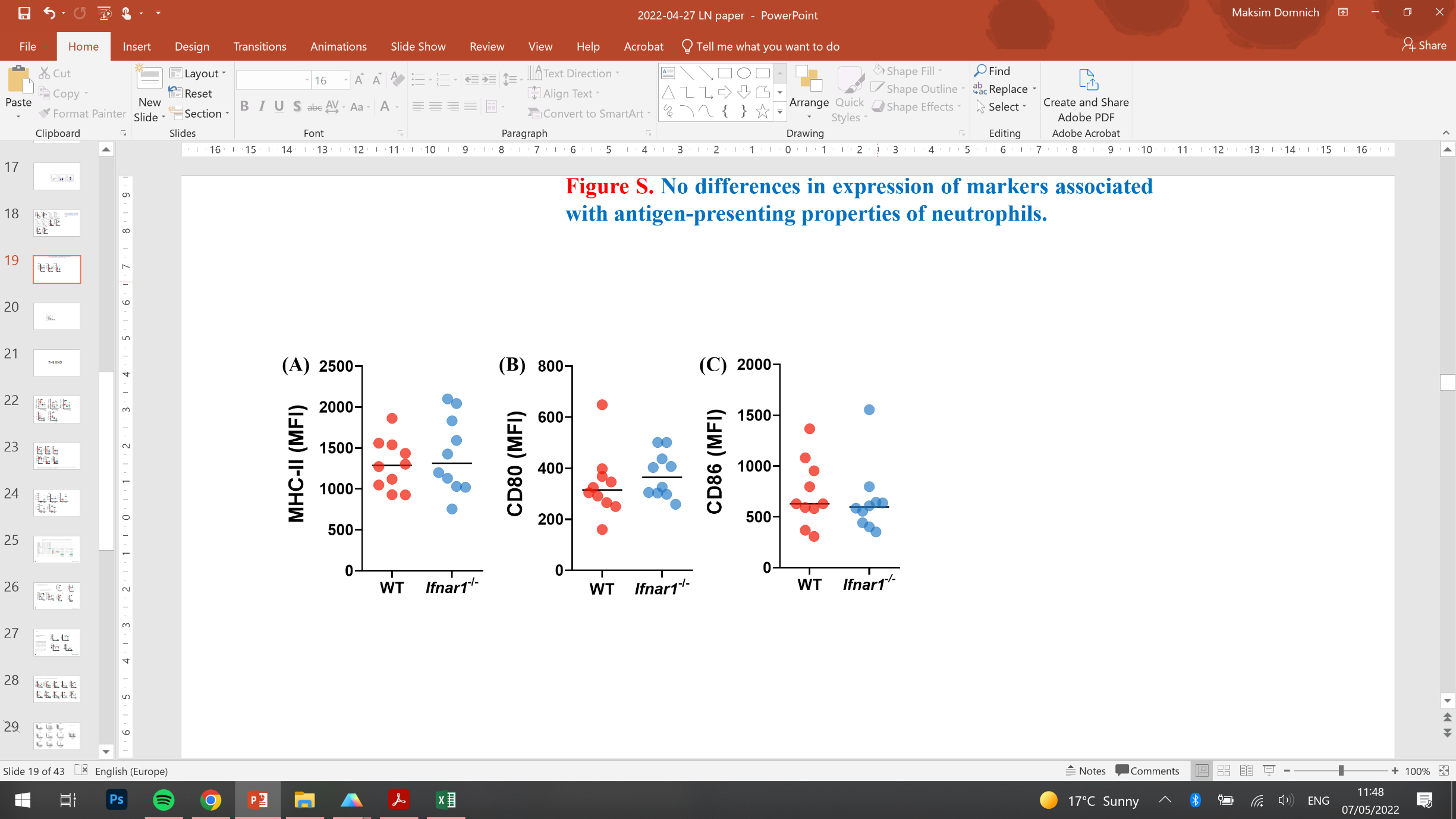


**Figure S8.** **No differences in expression of markers associated with antigen-presenting properties of neutrophils.** (**A-C**) The lack of significant differences in expression of markers responsible for T-cells activation (**A**) MHC-II, (**B**) CD80, (C) CD86. The MOPC cells were injected subcutaneously (1 x 10^6^ cells in 100 µl PBS) into the flank of WT (C57BL/6). On day 14 post tumor implantation mice were sacrificed, tumor-draining LNs were dissected, single-cell suspension was prepared, stained for mentioned markers and analyzed by flow cytometry.


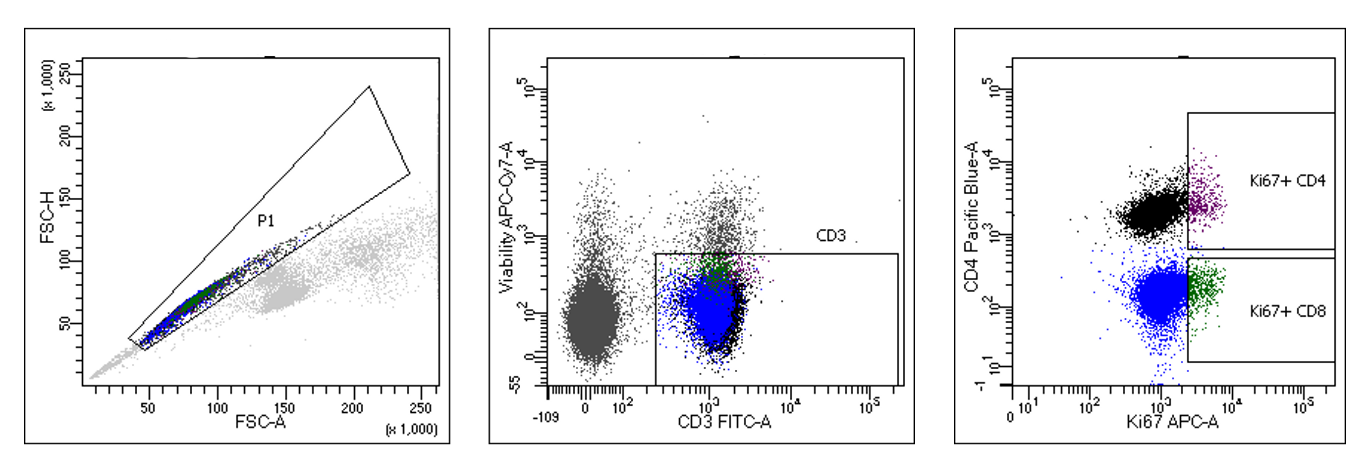


**Figure S9.** **Gating strategy for T-lymphocytes (Ki67 expression on single alive CD3^+^, CD3^+^CD4^+^ and CD3^+^CD8^+^ cells).** The MOPC cells were injected subcutaneously (1 x 10^6^ cells in 100 µl PBS) into the flank of WT (C57BL/6). On day 14 mice were sacrificed, tumor-draining LNs were dissected, single-cell suspension was prepared, stained for mentioned markers and analyzed according the showed flow cytometry analysis.
